# Network Neuroscience and Personality

**DOI:** 10.1017/pen.2018.12

**Published:** 2018-08-10

**Authors:** Sebastian Markett, Christian Montag, Martin Reuter

**Affiliations:** 1 Department of Psychology, Humboldt University, Berlin, Germany; 2 Department of Molecular Psychology, Institute of Psychology and Education, Ulm University, Ulm, Germany; 3 The Clinical Hospital of Chengdu Brain Science Institute, MOE Key Lab for Neuroinformation, University of Electronic Science and Technology of China, Chengdu, China; 4 Department of Psychology, University of Bonn, Bonn, Germany

**Keywords:** brain connectivity, connectome, resting-state fMRI, personality traits, conceptual nervous system

## Abstract

Personality and individual differences originate from the brain. Despite major advances in the affective and cognitive neurosciences, however, it is still not well understood how personality and single personality traits are represented within the brain. Most research on brain-personality correlates has focused either on morphological aspects of the brain such as increases or decreases in local gray matter volume, or has investigated how personality traits can account for individual differences in activation differences in various tasks. Here, we propose that personality neuroscience can be advanced by adding a network perspective on brain structure and function, an endeavor that we label personality network neuroscience.

With the rise of resting-state functional magnetic resonance imaging (MRI), the establishment of connectomics as a theoretical framework for structural and functional connectivity modeling, and recent advancements in the application of mathematical graph theory to brain connectivity data, several new tools and techniques are readily available to be applied in personality neuroscience. The present contribution introduces these concepts, reviews recent progress in their application to the study of individual differences, and explores their potential to advance our understanding of the neural implementation of personality.

Trait theorists have long argued that personality traits are biophysical entities that are not mere abstractions of and metaphors for human behavior. Traits are thought to actually exist in the brain, presumably in the form of conceptual nervous systems. A conceptual nervous system refers to the attempt to describe parts of the central nervous system in functional terms with relevance to psychology and behavior. We contend that personality network neuroscience can characterize these conceptual nervous systems on a functional and anatomical level and has the potential do link dispositional neural correlates to actual behavior.

## 1. Personality network neuroscience

Ever since the affective and cognitive neurosciences have embarked on their journey towards unraveling the biological basis of cognition, motivation/emotion, and behavior, new technologies and paradigms have shaped their path. One of the hallmark developments on the technological side was MRI. It enables researchers to non-invasively assess neural processes in the awake human brain (Turner & Jones, 2003). MRI is not perfect, just like any other scientific method, but it is of indisputable value for the study of the human brain (Turner, 2016). On the spatial level, MRI depicts anatomy of the living human brain with unprecedented spatial detail. On the functional level, it can keep track of ongoing activity in brain dynamics. Furthermore, MRI has been approved for use in healthy human research participants who volunteer their time for scientific enquiry. In this regard it is superior to other techniques like intracranial recordings, optogenetic imaging, or radioligand-based imaging which are either not approved for the use in healthy human volunteers, are not approved for use in humans at all, or are severely constrained due to the emission of harmful radiation. Since its introduction in the early 1990s, MRI has positioned itself as the methodological backbone of cognitive neuroscience and has provided marvelous insights into the neural foundation of psychological processes (Raichle, 2009).

On the paradigmatic side, the last 10 years have seen the rise of connectomics and network neuroscience as a new way to reason about the brain. The neologism connectome, introduced independently by Patric Hagmann and Olaf Spons in 2005 (Hagmann, 2005; Sporns, Tononi, & Kötter, 2005), combines the term “connection” with the suffix “-ome” which stands for “the whole class of something.” In analogy to the term genome (“gene” and “-ome”) which describes the entirety of and the organizational principle behind the genetic information of a species or an organism (Winkler, 1920), a connectome describes the organization of connections throughout a nervous system. The brain is a complex network whose intricacy can be apprehended on many different resolution levels. On a microscopic level, neurons sprout axons that find their way to the dense dendritic trees of other neurons where they connect via synaptic contacts. On a macroscopic level, the axons of thousands of neurons merge together in major white matter fiber bundles that project from one brain area to another. MRI technology can be applied to non-invasively map these fiber bundles in the human brain and to estimate from functional imaging data how information processing unfolds along these pathways. MRI connectomics has revealed that neural connections follow—despite all their complexity—certain organizational principles that enable efficient and goal-oriented information processing. Recent years have seen major advances in the field of network science. Sophisticated network modeling tools have been developed that are increasingly applied to brain connectivity data in order to unravel organizational principles and understand their relevance for psychological processes and clinical conditions.

Personality neuroscience as a new field of study within the cognitive and affective neurosciences has embraced neuroimaging, psycho-pharmacology, molecular genetics, and psychophysiological methods as its tools of choice to study the biological foundations of personality, and to derive explanatory models of individual differences (DeYoung & Gray, 2009). In the present contribution, we argue that neural network modeling techniques should step up to join the methodological pantheon of our field. We will outline how connectivity research can complement more traditional brain mapping approaches such as brain activation or simple morphological studies in assessing the biological bases of personality traits. To this end, we are going to introduce key concepts and findings of resting-state functional MRI (fMRI), diffusion MRI and basic ideas of network neuroscience, with the aim of promoting the application of network neuroscience in general, and MRI-based connectomics in particular, to the study of personality and as a basis of new types of personality theory (see sections 4 and 5). We will build our arguments mostly on studies investigating non-ability traits, even though similar ideas should in principle be also applicable to other aspects of individual differences.

## 2. Where nature meets nurture

Before we begin with introducing key concepts from brain connectivity, connectomics, and network science, we would like to briefly review the goals and aims of personality neuroscience. The key presumption of personality neuroscience is that a person cannot be understood without understanding their brain (DeYoung, 2010). Personality neuroscience therefore utilizes techniques from affective and cognitive neuroscience to relate brain processes to personality characteristics.

Personality psychology has developed sophisticated taxonomies to describe individual differences. Classificatory systems such as the Big Five model/five-factor model (Costa & McCrae, 1992; Goldberg, 1993), the six-dimensional HEXACO model (Lee & Ashton, 2008), or multi-factor hierarchical models (Cattell, 1965) mostly utilize self-report data to locate individuals in a multi-dimensional factor space. Although these models have been very successful in describing individual differences and also in predicting actual behavior from individual personality scores, they are mostly non-explanatory and allow for only limited insights into *why* people differ. The personality neuroscience approach which seeks to trace the neural implementation of personality factors in the brain is also not explicatory per se and often falls short of providing insights into causal mechanisms. It does, however, give a more detailed picture on how personality might work, and constitutes an attempt to unveil the neural foundation of personality that can be more easily traced to distal determinants of personality such as genetic and environmental influences (DeYoung, 2010). Population genetic studies can provide insights into such distal determinants of individual differences. A recent meta-analysis has compiled evidence from 2,748 twin studies of the last 50 years and has quantified genetic influences on personality at 49% on average (Polderman et al., 2015). This estimate suggests that roughly one half of the variance in individual differences in personality can be explained by genetic factors, while the other half should originate from environmental influences. Such work is based on the decomposition of covariance matrices. Despite its tremendous success in pointing towards broad classes of influencing variables, it still falls short of pointing towards explanatory pathways and mechanisms. Moreover, findings like the above from twin research too easily suggest for laypersons that nature and nurture are two distinct entities. But abundant research demonstrates that nature and nurture strongly interact (for a short overview, see Montag & Hahn, in press). Even when the recent advances in molecular genetics (for review, see Reuter, Felten, & Montag, 2016) and the utilization of genome-wide association designs and genetic complex trait analysis (Plomin & Deary, 2015) would lead to an exhaustive list of genetic polymorphisms responsible for individual differences in the personality domain (which is an utopia at the moment), we would still need to tackle the mechanisms by which these genetic variables interact with environmental influences and cause individual differences in behavior and behavioral dispositions. This problem also applies to environmental factors equivalently. Furthermore, new advances in the field of epigenetics suggest an impact of the environment on gene activity at a molecular level. Such gene-environment interactions are currently studied by analyzing methylation patterns in promoter regions of genes and histone modification (Zhang & Meaney, 2010). Personality neuroscience, with its focus on neural processes, will be of much value by describing personality and individual differences at the brain level and hence providing intermediate mechanisms that bridge between personality and its more distal influences such as molecular genetics, gene by environment effects and the epigenome. This idea is also currently utilized in the field of psychiatric genetics that faces quite similar challenges in linking genetic variation to complex behavioral traits and individual differences (Meyer-Lindenberg & Weinberger, 2006).

In order to be successful in explaining individual differences and personality, we need to ask how to best derive such intermediate neural models of personality. Here, we can get inspiration from behavioral biology. In the 1980s, behavioral biologists and geneticists published a complete list of all neurons and their synaptic connections of the nematode *Caenorhabditis elegans*, a widely studied model organism in biology (Emmons, 2015; White, Southgate, Thomson, & Brenner, 1986). This roundworm possesses only a small number of neurons (around 300) that form around 5,000 chemical synapses. The rationale behind this effort was straightforward: synaptic contacts between single neurons are established during learning and can thus depend on experience (Kandel & Schwartz, 1982). Genetic mechanisms are similarly involved in synaptic plasticity and long-term potentiation (Alberini, 2009). Identifying a neural circuit with relevance for behavior and then studying its genetic and experience-dependent determiners thus qualify as holistic approach with potential to go way beyond mere description (Bargmann & Marder, 2013).

Studying nematode behavior and neural connectivity has at best only limited potential for understanding human individual differences. The research strategy, on the other hand, could prove very fruitful, and clearly the study of *C. elegans* and *Aplysia californica* led to groundbreaking insights (for an overview, see Hawkins, Kandel, & Bailey, 2006). At present, however, *C. elegans* is the only organism with a completely recovered network map at the cellular level. Similar efforts in other model organisms such as Drosophila or the mouse are still pending, mainly because of the complexity of their nervous systems (Schröter, Paulsen, & Bullmore, 2017). Fortunately, such detail in mapping neural connectivity is not required to assess organizational principles of brain networks. White matter fiber tracts consist of thousands of single axonal connections that share an area of origin and a projection site. A large number of myelin sheets that insulate the axons affect signals that can be picked up by MRI scanners. Macro-level brain connectivity in the form of white matter fiber tracts can be revealed by modern MRI technology without detailed knowledge of micro-level connectivity on the level of single neurons (Sporns, 2013).

Previous work has successfully demonstrated that experienced-based changes in human behavior and ability are accompanied by changes at the level of brain networks (Lewis, Baldassarre, Committeri, Romani, & Corbetta, 2009; Scholz, Klein, Behrens, & Johansen-Berg, 2009), and that individual differences in brain networks show associations with genetic variation (Markett et al., 2016a, 2017a). Nature and nurture affect individual differences and personality. Environmental influences reach the brain through all afferent projections, mainly from sensory sources. Each cell within the brain contains a complete copy of the genome and genetic information is constantly transcribed with marked effects on neural processing. It is within the human brain where nature and nurture meet. Models of the brain and its functions should thus provide an ideal background for the study of mental faculties such as human personality.

## 3. Personality traits and their neural implementation

Even though most definitions of personality emphasize a holistic perspective on individual differences, personality neuroscience has so far mostly focused on the neural implementation of personality traits (DeYoung, 2010). In our present line of argumentation, we also focus primarily on personality traits, while acknowledging that this captures only parts of human personality.

The trait definition by Gordon Allport, one of our field’s early eminent trait theorists, views personality traits as neuropsychological systems with the purpose of making a variety of stimuli functionally equivalent, in order to trigger a consistent and meaningful response (Allport, 1937). Functional equivalence means that a “common ground” of a class of stimuli is extracted, in order to initiate an appropriate response. As an example to illustrate this rather abstract definition, consider the emotion anxiety. There is a fair deal of stimuli that have the potential to be perceived as frightening. This can range from social situations such as public speech, to approach-avoidance conflicts such as taking an important exam, and to situations with high uncertainty and potential danger such as a dark alley at night. These situations might appear as entirely different things at first glance but can very well trigger a feeling of anxiety. The trait concept assumes that distinct circuitries in our brains (the neuropsychological systems from the definition) are dedicated to extract the frightening/worrying components from the stream of incoming information and initiate a consistent response that might involve a reappraisal of the situation, careful exploration, and rumination, or—in case of an imminent threat—flight, fight, or freezing (Gray & McNaughton, 2000). Individual differences come into play by the assumption that the reactivity or sensitivity of a given neuropsychological system varies across people. People who are more of the anxious type react with a stronger or more frequent anxiety response than less anxious people. The trait concept can thus account for at least three aspects of behavior: (a) that entirely different situations can lead to a similar affective or motivational reaction, (b) that people differ in the perception of external stimuli as positive or threatening, and that (c) the strength and even direction of the reaction to these stimuli is marked by individual differences. The trait definition is of course not limited to the description of dispositional differences in anxiety but applies to other aspects of personality such as extraversion or openness to experience as well.

This definition of a personality trait follows a neo-behavioristic line of thought. Neo-behaviorism positions that behavior can be best described in terms of a S→O→R equation, where S denotes a Stimulus, R an overt or covert reaction, and O an organism. We use the prefix *neo-* with the term *behaviorism* to emphasize that the trait definition does neither imply that the organism is a black box that is inaccessible to rigorous scientific examination, nor that all behavior and behavioral disposition are solely the product of learning and conditioning processes. By emphasizing the organism variable in the form of neuropsychological systems as the main substrate of the trait, the brain is brought into the focus of trait research. Traits are not only statistical abstractions in the imagination of psychometricians, but have a hard-wired biological analogue in the physical world that can be localized and assessed by neuroscientific methodology. This conceptualization of traits as neuropsychological systems does not imply 1:1 correspondence between single brain structures and personality traits. Throughout the central nervous system, brain structures are wired together into systems with certain functional roles. One central assumption of personality neuroscience is the existence of distinct brain systems for distinct traits. This functional perspective on brain systems is also reflected in the idea of the conceptual nervous system that seeks to explain the brain on the conceptual rather than the anatomical level (Gray, 1971; Gray & McNaughton, 2000).

Another corollary of the trait definition is the emancipation of the trait concept from trait-relevant stimuli and the organism’s behavioral response. Even though traits have the purpose to operate on environmental stimuli in order to trigger contingent responses, the neuropsychological systems that constitute the traits exist independently from the outside world (Mischel & Shoda, 1995). A key feature of personality traits is their temporal stability over longer stretches of an individual’s lifespan (Edmonds, Jackson, Fayard, & Roberts, 2008; Specht, Egloff, & Schmukle, 2011). An individual with a certain trait characteristic should respond similarly over many instances of stimulus presentation over the period of several months if not years. Examining the interaction of the neuropsychological trait system and an environmental stimulus is therefore as informative for personality neuroscience as the examination of neural systems in the absence of stimulation. We emphasize this point because it is often overlooked in traditional personality neuroscience research, and because we believe that tools from network neuroscience can make a genuine contribution in this regard.

The majority of functional imaging studies on personality so far have mainly examined the interaction of trait expression and stimulus processing. The common paradigm entails the recording of neural activity in response to a stimulus and the assessment of the extent to which the strength of the neural response depends on individual trait levels. The involved stimuli can range from the simple passive viewing of emotional faces (e.g., Canli et al., 2001; Reuter et al., 2004) to complex naturalistic scenarios with actual behavioral relevance for the participants (e.g., Mobbs et al., 2007), and various personality traits such as neuroticism (Haas, Omura, Constable, & Canli, 2007), extraversion (Cohen, Young, Baek, Kessler, & Ranganath, 2005), or self-transcendence (Montag, Reuter, & Axmacher, 2011) have been examined. This research strategy has led to a substantial body of research on the neural correlates of individual differences. Taken together, the available evidence unequivocally confirms that neural systems differ in their reactivity to external stimulation depending on a person’s personality (see Kennis, Rademaker, & Geuze, 2013 for review, and Calder, Ewbank, & Passamonti, 2011, for a coordinate-based activation likelihood estimation meta-analysis). This focus, however, does not distinguish between stimulus processing and the neuropsychological trait system itself. Following the idea that traits correspond to stable neuropsychological systems that exist in the human brain independently from the outside world, neural counterpart of personality trait should be reflected in intrinsic properties of the brain. For the longest time, morphological studies of the brain have been the methodological approach of choice to assess such stable, stimulus-independent neural correlates of behavioral dispositions (e.g., DeYoung et al., 2010; Liu et al., 2013; Riccelli, Toschi, Nigro, Terracciano, & Passamonti, 2017; for a meta-analysis, see Mincic, 2015). Voxel-based morphometry studies, in particular, provide detailed insights into neuro-structural correlates of personality traits and have shown that brain regions with trait-dependent activation profiles also show trait-dependent differences in their morphology (Omura, Todd Constable, & Canli, 2005, but see also Liu et al., 2013 for replication issues). Within biological systems it is often said that function follows structure (Kristan & Katz, 2006), and the study of structural personality correlates should thus be informative for the understanding of functional alterations. The relationship between macroscopic morphology and neural activity, however, is not straightforward and is very likely to involve many intermediate steps. It will be necessary to consider additional aspects of brain structure and function in order to fully map various neuropsychological trait systems. Moreover, efforts to bring together structural and functional brain imaging data in personality neuroscience are scarce, until now.

Brain connectivity could represent an important step forward to a more comprehensive understanding of the neuroscientific basis of human personality. Early biologically oriented personality psychologists have noted that it is always several brain areas (and not a single one) that underlie fundamental personality traits. The relevance for a trait system might not become apparent from anatomy alone. Jeffrey Gray has coined the term “conceptual nervous system” to describe neural systems from the perspective of their functional and behaviorally relevant purpose (Gray, 1971; Gray & McNaughton, 2000). A conceptual nervous system implies several connected brain areas. From this perspective, Gray can be seen as an early proponent of a personality network neuroscience. The notion of “brain networks” is mostly implicitly assumed in neuroimaging studies, but not explicitly assessed in terms of connectivity between multiple brain areas. The connectome paradigm offers the methodological toolbox to advance the field towards unraveling the connectivity patterns underlying personality traits. Resting-state fMRI allows us to examine the intrinsic functional architecture of the human brain, independent from external stimulation. We are going to introduce this and other techniques of relevance in connectomics in the following.

## 4. Connectivity and networks: The connectome paradigm

Connectomes are network maps of brain connectivity. In general, connectomes can be studied on different scales, and the resolution level determines what entity is considered a connection and what entities are tied together by connections (Sporns, 2016). In studies on human brain networks, connectivity estimates are usually derived from neuroimaging data and the finest resolution level is thus restricted to single voxels, although a less-fine-grained resolution at the level of larger brain areas is more common.

**Figure 1:**
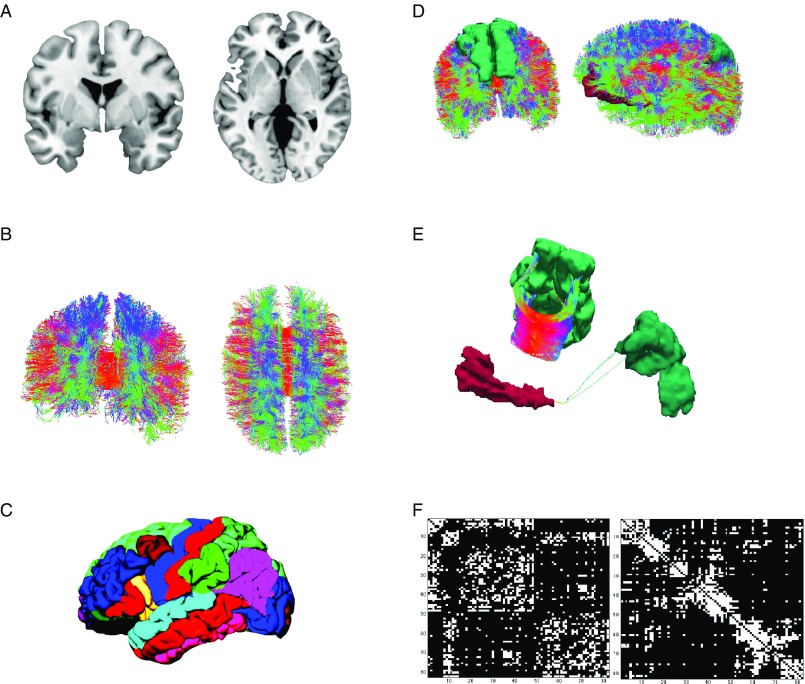
Illustration of the workflow of connectome reconstruction. A connectome combines information from cortical gray and white matter (A). Diffusion imaging and computational reconstruction methods are applied to obtain a detailed map of white matter fiber connections (B). The cortical ribbon is parcellated into a set of non-overlapping regions of interest, (C) illustrates Freesurfer’s Desikan-Killiany atlas. Connectome reconstruction combines the information from (B) and (C) and determines whether two ROIs are touched by any white matter fibers or not. (D) Shows two examples: the left and right superior frontal gyrus (left) are densely connected (E, left) while the left medial orbitofrontal and superior parietal gyrus (D, right) are only sparsely connected (E). Results can be displayed in a connectivity matrix. Rows and columns are regions of interest from the whole brain parcellation. Matrix elements indicate the presence (white) or absence (black) of a connection (F). The left matrix groups brain regions according to hemisphere and cortical vs. subcortical, the right matrix according to their allegiance to network modules.

When looking at gross brain anatomy, it becomes apparent that the location of gray matter is restricted to the cortical ribbon and subcortical structures (see Figure 1A). Neurons in layer three of the cortical ribbon grow axons that leave cortical gray matter and descend to white matter areas before making contact with neurons in layer one or two of the cortical sheet in a distal part of the brain. Beginning with the work by early neuroanatomists, attempts were made to delineate the cortical ribbon into distinct brain regions based on their cytoarchitecture (Brodmann, 1909; von Economo & Koskinas, 1925), or other properties such as gyri- and sulcification (Tziourio-Mazoyer et al., 2002), thickness of the cortical gray matter sheet (Fischl & Dale, 2000; Fischl et al., 2004, see Figure 1C), or the synthesis of multimodal brain imaging techniques (Glasser et al., 2016). When assembling a connectome map, one of such whole brain parcellation schemes is chosen and for each pair of brain regions it is determined whether they are connected or not (Hagmann et al., 2010, see Figure 1D). Connectivity is either assessed on the structural or functional level. This distinction is crucial for the connectome paradigm.

The study of structural connectivity in the form of anatomical white matter fiber projections has become feasible with the advent of diffusion-weighted MRI (Le Bihan et al., 1986), its refinement as diffusion tensor imaging (DTI; Basser, Mattiello, & LeBihan, 1994) and the introduction of computer-assisted fiber-tracking (Basser, Pajevic, Pierpaoli, Duda, & Aldroubi, 2000). In DTI, water diffusion in biological tissue is described by a three-dimensional tensor model. DTI exploits the shape of the diffusion tensor which is affected by factors that restrict water diffusion in a sample, such as the fat content of myelin sheets that insulate axonal projections. Myelin sheets wrap around axons and thus restrict water diffusion along white matter pathways. Each voxel’s principle diffusion direction can be obtained from its diffusion tensor and computational approaches can be used to reconstruct major white matter projections in the brain (see Figure 1B). White matter tractography is by no means a new development in our field. The connectome paradigm, however, builds up on these developments and provides an integratory framework for the evaluation of white matter tracts throughout the entire brain. A shortcoming of the DTI method is its inability to detect directionality. From DTI alone, it cannot be inferred whether one regions projects to the other or vice versa. At present, this can only be achieved by means of invasive tract tracing and is therefore not applicable in human research participants.

**Figure 2:**
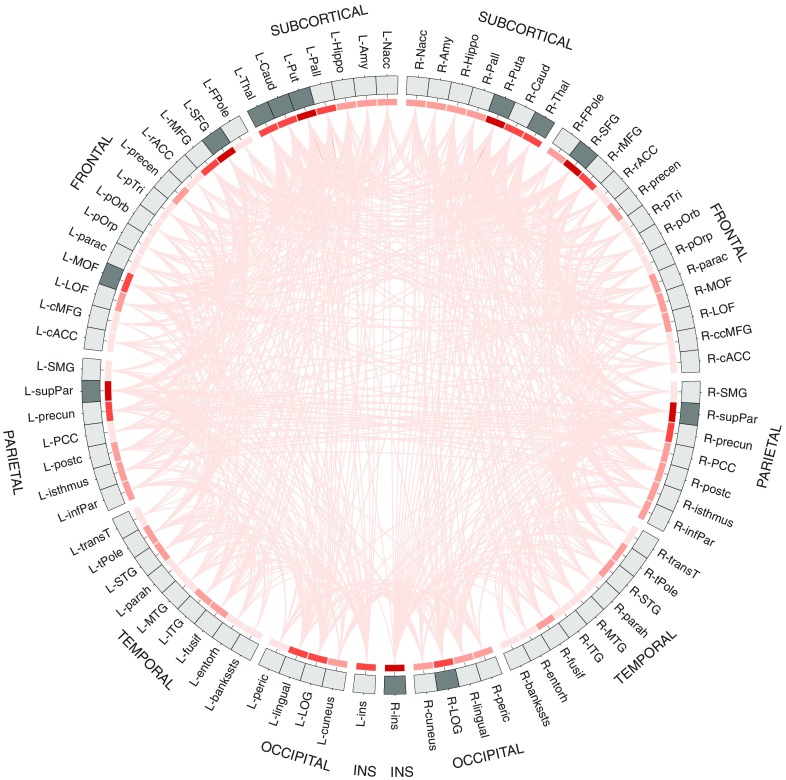
Circos plot (Krzywinski et al., 2009) depiction of a structural connectome. In total, 82 brain regions of Freesurfer’s Desikan atlas are ordered according to hemisphere and lobe, and arranged on a circle. The red heat map illustrates degree centrality, a measure that quantifies the brain region’s number of connections (darker shades indicate higher degree). The brain areas in dark gray are the brain regions with the highest degree (top 15%) that qualify as possible hub regions. Data are taken from Markett et al. (2017b).

Results from connectome fiber tracking can be depicted in a matrix that lists brain regions row- and columnwise, where matrix elements indicate whether two brain regions are connected or not (see Figure 1F). Matrix elements can also give connection weights, reflecting the absolute or relative strength of the connection (e.g., based on streamline count or on summary measures of fiber tract integrity such as fractional anisotropy). Connectome matrices from diffusion MRI data are always symmetrical because it is not possible to derive information on the directionality of structural connectivity from this data source. Figure 2 illustrates a fully assembled connectome as a circos plot.

**Figure 3:**
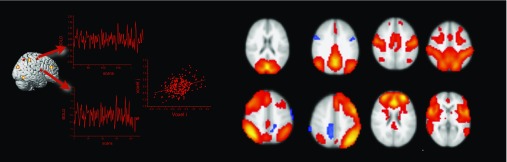
Functional connectivity from blood oxygen level dependent (BOLD) functional magnetic resonance imaging is estimated by correlating the extracted BOLD time series from regions of interest or single voxels (left panel). The right panel shows prominent resting-state functional connectivity networks as revealed by independent component analysis (data courtesy of the authors). The networks in the top row correspond from left to right to the visual, the default mode, the somatomotor, and the dorsal attention network. The networks in the bottom row correspond to the left and right fronto-parietal, the frontal, and the salience network. All networks are displayed in radiological convention (left is right).

Brain regions exchange information along the elements of the neuro-structural white matter scaffold (Park & Friston, 2013). The synchronization of neural activity from different brain regions is commonly interpreted as evidence for a functional coupling of these regions (Friston, Frith, Liddle, & Frackowiak, 1993). The assessment of functional connectivity on the grounds of fMRI is thus correlational, and the most common approach is to compute simple linear correlations between blood oxygen level dependent (BOLD) time series data from two or more brain regions (see Figure 3). For BOLD fMRI, whose temporal precision is restricted by the sluggishness of brain hemodynamics, simple linear correlations are sufficient. Imaging modalities with a higher temporal resolution than BOLD fMRI, however, can require more sophisticated coherence measures. The use of functional connectivity data in connectome studies has been tremendously advanced by resting-state fMRI (Smith et al., 2013). Resting-state fMRI is an experimental neuroimaging protocol where BOLD activity is recorded from the brains of research volunteers who do not engage in a particular task (Fox & Raichle, 2007). In contrast to brain activation studies that aim at the isolation of neural correlates of circumscribed cognitive and emotional processes, resting-state fMRI assesses intrinsic, non-stimulus-dependent neural activity. In a pioneering resting-state fMRI study, Biswal, Zerrin Yetkin, Haughton, and Hyde (1995) reported highly synchronized brain activity in brain regions that are commonly co-activated during simple motor tasks. Because this synchronization occurred in the absence of any motor behavior or motor planning, it was interpreted as an intrinsic somatomotor network (see Figure 3). Subsequent studies confirmed the robustness of the finding and also provided evidence for other intrinsic connectivity networks such as the default mode network (Greicius, Krasnow, Reiss, & Menon, 2003), the insular-opercular network (Seeley et al., 2007), the fronto-parietal network (Fox, Snyder, Vincent, Corbetta, & Van Essen, 2005), and networks in visual areas (for review, see van den Heuvel & Hulshoff Pol, 2010). Depending on the analysis method and resolution parameters, 7–12 robust intrinsic resting-state connectivity networks are commonly found. It has been shown that these networks correspond to task-related brain activity, in a way that brain regions that connect together at rest activate together within and across experimental tasks (Gordon, Stollstorff, & Vaidya, 2012; Smith et al., 2009). In comparison to task fMRI, resting-state activity places a much higher burden on the brain’s total metabolism budget (Raichle, 2006), which indicates that the maintenance of intrinsic activity is biologically costly which is usually a sign of functional importance. In summary, the key finding of resting-state fMRI is that single brain regions synchronize their activity even in the absence of external stimulation. By applying multivariate statistics, it can be shown that this synchronization is organized into large-scale brain networks that together form the *functional connectome*. It has been shown that the functional connectome relates closely to its structural counterpart (Honey et al., 2009; Horn, Ostwald, Reisert, & Blankenburg, 2014). Both types of connectivity, however, are non-redundant and provide unique insights into the network level architecture of the human brain. Consider a train network as an analogy: in this analogy, structural connectivity between brain regions would represent railway tracks between various train stations. The train schedule, on the other hand, would be akin to functional connectivity. The physical railway network places major constraint of the train schedule for sure, however, both types of information will be relevant for railway engineers, traffic control, and passengers alike.

**Figure 4:**
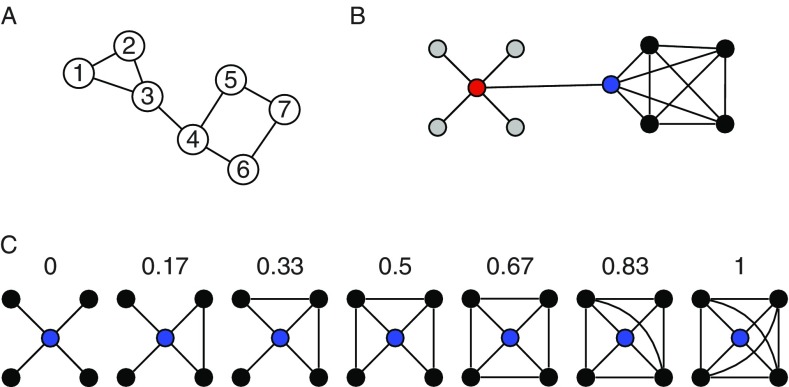
Toy networks for the illustration of measures from network theory. The global efficiency of a network can be quantified by its characteristic path length (CPL). CPL is the average of all shortest path length in a network. The shortest path between any two nodes in a network equals the minimum number of edges that have to be transversed to reach one node from the other. In the network in (A), the shortest path equals 1 between node 1 and node 2, equals 2 between node 1 and node 4, and 4 between node 1 and node 7. The CPL of the network is 2.0476. The toy graph in (B) illustrates degree centrality and betweenness centrality. Degree centrality of a node equals the number of its connection to other nodes. The gray nodes have a degree of one, the black nodes a degree of four, and the blue and red node a degree of 5. In general, it is assumed that high degree nodes are more important for the network as a whole. Betweenness centrality is a further centrality measure that can capture different information on the importance of a node. Betweenness reflects the amount of shortest paths between any two nodes that travel through a given node. The red node, for instance, has a higher betweenness than the blue nodes (even though they have the same degree). All shortest paths between any gray and any black node travel through the red and the blue node, however, all shortest paths between any two black nodes travel through the red but not through the blue node, hence its higher betweenness. The networks in (C) illustrate the clustering coefficient, which is regarded a measure of local efficiency. The numbers indicate the clustering coefficient for the red node in the network. The clustering coefficient gives the ratio of neighboring nodes that share a connection themselves over the total number of connections that neighbors could share. In the first graph, none of the black nodes (the neighbors) share a connection, hence the clustering coefficient of 0. In the last network, all neighbors are directly connected to each other (six connections between neighbors), hence the clustering coefficient of one.

The connectome paradigm has gone further than just providing a method toolbox for the assessment of structural and functional connectivity. More abstract organizational principles of brain networks can be described by the utilization of mathematical network theory. In network theory, a network consists of a set of network nodes such as brain regions that are fully or partially connected by a set of links such as structural and/or functional connectivity (Albert & Barabasi, 2002). By applying transformations to the connectome matrix, a set of key observations on brain networks have been observed: human brain networks have a relatively sparse connection density which means that most brain regions merely maintain direct connections to a few other brain regions (Hagmann et al., 2007). Only a small amount of brain regions maintain many connections. These regions serve as network hubs in the brain and are crucial for maintaining a highly efficient information exchange across subnetworks (de Reus & van den Heuvel, 2014; Hagmann et al., 2008; van den Heuvel & Sporns, 2013, see Figure 2). The organization of brain networks into local subnetworks and a set of integratory hubs ensure both local and global efficiency in information flow, an organizational principle described as “small-world structure” (Sporns & Zwi, 2004). Small-world networks are characterized by a high connection density between neighboring nodes (characterized by high clustering coefficients, see Figure 4C), and short communication paths between distant network nodes that enable quick information exchange across the entire network (characterized by short characteristic path length, see Figure 4A).

Several studies have sought to relate individual differences in such anatomical and neuro-functional organization principles of human brain networks to personality. We will review some of these findings in the next section.

## 5. Network level correlates of personality

The easiest and most basic connectomic studies make use of the seed method. This method defines a starting point in the brain (the seed region) and then examines all structural or functional connectivity originating from this seed. Several studies have focused on the relationship between personality traits and amygdala connectivity, presumably based on the prominent role of the amygdala region for affective processing. Aghajani et al. (2013) report differential patterns of functional connectivity patterns originating from the amygdala depending on neuroticism and extraversion scores. Li, Qin, Jiang, Zhang, and Yu (2012) have provided a more fine-grained perspective on amygdala connectivity by linking harm avoidance—a measure related to neuroticism—to functional connectivity patterns of different amygdala subregions. Distinguishing between different amygdala subregions is important as the amygdala consists of different nuclei that play different roles in generating behavior (Ledoux, 2003). Even though it is difficult to image the medial temporal region due to frequent signal distortions (Olman, Davachi, & Inati, 2009), it has been shown that different amygdala subregions have distinct functional connectivity profiles at rest with unique phenotypical associations (Eckstein et al., 2017; Roy et al., 2009). Amygdala functional connectivity was also shown to relate to trait anger (Fulwiler, King, & Zhang, 2012) and the SADNESS trait from the Affective Neuroscience Personality Scales (Deris, Montag, Reuter, Weber, & Markett, 2017; for the importance of Panksepp’s Affective Neuroscience Theory for personality neuroscience, see Montag & Panksepp, 2017a, 2017b). Another seed-based functional connectivity study from our group has used the anterior insula as seed to show insula-centered connectivity differences in harm avoidance (Markett et al., 2013). The most comprehensive resting-state functional connectivity study on personality to date has used multiple seed regions placed throughout central areas in major resting-state networks and reported wide-spread associations between all Big Five personality traits (Adelstein et al., 2011). Based on their findings the authors concluded that personality is reflected in the brain’s intrinsic functional architecture.

The link between neuroticism-related personality traits and amygdala as well as insula connectivity has been corroborated by structural connectivity studies. Westlye, Bjørnebekk, Grydeland, Fjell, and Walhovd (2011) have linked harm avoidance to the integrity of cortico-limbic white matter tracts such as uncinate fasciculus which connects mid-temporal structures including the amygdala to anterior regions. In a similar vein, Montag, Reuter, Weber, Markett, and Schoene-Bake (2012) report a link between a composite measure of trait anxiety and the structural integrity of the uncinate fasciculus as well. And Baur, Hänggi, Langer, and Jäncke (2013) report that structural connectivity between the amygdala and the anterior insula indexes trait anxiety. The idea that mid-temporal to anterior connectivity is an essential brain connectivity level correlate of trait anxiety and neuroticism has also been proposed by Montag, Reuter, Jurkiewicz, Markett, and Panksepp (2013) in a systematic review of the structural neuroimaging literature on anxiety.

Personality network neuroscience promises an integrative, network level account of brain–personality relationships. The connectivity studies reviewed so far have exclusively focused on single brain regions and single brain connections in their study of personality traits. However, they do point towards one large-scale brain network called the insular salience network (Seeley et al., 2007). This network centers around the anterior insula, includes the anterior cingulate, parts of the basal ganglia, and cortical regions along the operculum, and receives input from the amygdala via the uncinate fasciculus. In a recent report from our group, we examined whether information processing efficiency in the insular salience network as a whole relates to trait anxiety (Markett, Montag, Melchers, Weber, & Reuter, 2016b). We modeled the entire insula network as weighted graph and computed the network’s characteristic path length as a measure of network efficiency. Characteristic path length is a summary measure of all shortest-communication connections between all network nodes in a given network (see Figure 4A). Using resting-state functional connectivity data, we found that harm avoidance as an index of trait anxiety related negatively to the insula network’s information exchange efficiency. Such system-level approaches have gained popularity in recent years. Beaty et al. (2016) have used a similar strategy to ours to link efficiency of the default mode network to openness to experience. Bey, Montag, Reuter, Weber, and Markett (2015) analyzed functional connectivity between large-scale functional brain networks and showed that functional connectivity of the insular network as a whole relates to individual differences in the susceptibility to cognitive failure. Toschi, Riccelli, Indovina, Terracciano, and Passamonti (2018) used a similar approach to large-scale functional brain networks, but applied graph–theoretical assessment of between-network connectivity to link organizational features of the functional connectome to the Big Five personality traits. In this work, conscientiousness was related to local aspects of the fronto-parietal and the default mode network. And Kyeong, Kim, Park, and Hwang (2014) examined functional connectivity between all cortical and subcortical brain regions in a whole brain parcellation scheme and showed that the intrinsic organization of the functional connectome into large-scale network is different depending on approach- and avoidance-related personality traits. Whole brain connectomic data were also analyzed by Gao et al. (2013). In a network analysis of functional connectivity between 90 brain regions from the automatic anatomical labeling atlas, they found that extraversion relates positively to the global clustering coefficient, a measure of the clustering in a network (see Figure 4C). Brain networks of more extraverted people showed a higher local clustering of brain connectivity across the entire brain, indicating that such a global organization property carries information of personality. In addition to this global association, the authors also report relationships between both neuroticism and extraversion and the betweenness centrality of several brain regions (see Figure 4B). Betweenness centrality is a network measure that quantifies how many shortest communication paths within a network run through a given brain node. In other words, the metric uses whole brain connectivity information to infer a single region’s importance to network communication. A highly interesting aspect of the Gao et al. (2013) paper is the attempt to predict individual personality scores from topological aspects of the brain network. A separate prediction model was set up for each participant by estimating a regression function from the data of all other participants (leave-one-out-validation). Prediction accuracies for extraversion was 11.4% and 21.7% for neuroticism across all participants. These numbers are of course not very high, but given that only a subset of possible network aspects were sampled in a cross-sectional design, it shows that network aspects of the brain do carry relevant information on personality. A further whole brain connectome study by Servaas et al. (2015) gives a detailed perspective of the neurotic brain. In their study, high neuroticism was associated with weaker functional connections throughout the entire brain. In consequence, functional subnetworks were less clearly delineated and the whole brain network had a higher resemblance to a network with random connection wiring. The aforementioned studies on whole-brain connectivity illustrate that global properties of the brain’s connectivity architecture relate to personality traits, and emphasize the importance of looking beyond connectivity of single brain regions such as the amygdala, or single functional brain networks such as the salience network. The discovery of large-scale connectivity networks (see Figure 3) might suggest that each of these network could have a circumscribed functional role, or might present a trait system on its own. Mounting evidence suggest that this is not the case, as behavior and behavioral dispositions are realized by the joint effort of several functional networks (Barrett & Satpute, 2013; Sylvester et al., 2012; Toschi et al., 2018; Touroutoglou, Lindquist, Dickerson, & Barrett, 2015). Personality neuroscience will need to understand the interplay between brain areas and between large-scale brain networks in order to derive complete network accounts of a neuropsychological trait systems.

At present, the literature on the relationship between personality and network aspects of brain structure and function places a strong emphasis on neuroticism and avoidance-related personality traits, and to a lesser extent on extraversion and approach-related traits. This focus might reflect a research bias in favor of clinically relevant personality traits, but could also result from more straightforward relationships between these personality traits and aspects of brain organization that are easily assessable with the field’s current methodological toolbox (Bjørnebekk et al., 2012; Markett, Montag, & Reuter, 2016c). Neuroticism and trait anxiety seem to be linked to several aspects of brain connectivity, ranging from associations between single brain areas and their connections, via the organization of single brain networks, to organizational principles of whole-brain connectivity. We believe that the evidence available thus far is encouraging for further investigations into the connectomics of personality. Further research will also want to reconcile the mostly MRI-based connectomic findings with research from other imaging modalities. There is a large body of resting-state EEG studies that has repeatedly demonstrated a relationship between frontal asymmetries in the alpha band and approach- and avoidance-related personality traits (Davidson, 2004; Wacker, Chavanon, Leue, & Stemmler, 2008). Frontal asymmetries are commonly interpreted in terms of hemispheric dominance. This could actually reflect the functional interplay of bilateral brain networks such as the fronto-parietal network or the insular salience network. Most cortical regions show a high degree of synchronization with their contralateral counterpart in resting-state BOLD time series (Zuo et al., 2010), and a first report suggests a relationship between novelty seeking, an approach-related personality trait, and the lateralization of functional connectivity of the anterior insula (Kann, Zhang, Manza, Leung, & Li, 2016).

As a last point, we want to come back to our initial proposal that network maps of the brain might prove useful for the evaluation of distal influences on personality such as genetic and environmental influences. An important first step would be to establish not only correlates between brain networks and personality but also links between brain networks and genetic variation and activity, and between brain networks and environmental influences. Studies have started to examine the relationship between the organization of brain connectivity and cortical gene expression (Forest et al., 2017; Romme, de Reus, Ophoff, Kahn, & van den Heuvel, 2017; Wang et al., 2015) or between brain connectivity and genetic variation (Markett et al., 2016a, 2017a, 2017b). Other studies have focused on experience-dependent changes in brain connectivity that might reflect Hebbian plasticity (Dosenbach et al., 2007; Lewis et al., 2009; Taubert, Villringer, & Ragert, 2012). We believe that this first evidence is encouraging for future investigations and justifies cautious enthusiasm that the connectome paradigm and a personality network neuroscience can make contributions to personality psychology that go beyond simple brain-personality associations.

## 6. Summary and outlook

In the present contribution, we have argued that network modeling of brain connectivity data has considerable potential for personality neuroscience. We have iterated the theoretical foundations of personality neuroscience: that studying the human brain in the context of personality research is more than another empirical layer of observation and can be very informative for reconciling genetic and environmental influences on personality. We have used the trait definition to argue that more traditionally designed brain activation assays and simple morphological studies are by themselves not sufficient to fully map and understand the neural circuitry underlying personality traits in the form of conceptual nervous systems. We have introduced the connectome paradigm, described basic methodological approaches to structural and functional connectivity, and their use in deriving network models of the human brain. After these theoretical considerations, we have provided a few examples of personality network neuroscience studies that have been published in the last few years.

We hope that our readers found our argumentation compelling and our introduction into personality network neuroscience appealing. We hope that our ideas might inspire a road map for the quest towards unraveling the causal basics of human personality. This being said, we have to acknowledge the many challenges and open questions remain that will need to be solved and answered in the future.

One question concerns the stability of the relationships with brain networks and personality traits. As personality traits are defined as relatively stable behavioral dispositions, any true relationship between personality traits and network aspects of the brain should show a similar degree of temporal stability. The human brain is subject to plastic changes by experience and learning (Kolb & Whishaw, 1998), which points to the question of temporal stability in structural and functional networks. Studies suggest stability of certain aspects of brain connectivity (Cao et al., 2014; Poppe et al., 2013) while other studies have reported a certain amount of plasticity in brain connectivity (Scholz et al., 2009). Lifespan studies on personality indicate that personality changes over the lifespan, particularly during maturation from adolescence to early adulthood (Blonigen, Carlson, Hicks, Krueger, & Iacono, 2008; Roberts, Caspi, & Moffitt, 2001), and that some changes in traits depends on the personality profile at younger age (Donnellan, Conger, & Burzette, 2007; Lönnqvist, Mäkinen, Paunonen, Henriksson, & Verkasalo, 2008). Longitudinal studies on personality and brain connectivity are still outstanding. Such longitudinal efforts should clarify which aspects of brain networks show a similar stability with personality traits and change accordingly when personality changes over the lifespan (Specht et al., 2011).

A second question is the disentanglement of states and traits. Cole, Bassett, Power, Braver, and Petersen (2014) report that the brain-wide organization of functional connectivity into a set of functional network modules is highly similar across different cognitive and affective task states, and resembles the modular structure of the resting state. On the other hand each task is characterized by subtle yet specific changes to the modular network structure. This work points at specific trait-like and state-like aspects of brain networks whose contribution to the state-trait distinction in personality science has to be assessed in future work. First studies on state-trait distinctions in brain networks indicate that changes in the brain’s intrinsic architecture across several states depend on individual differences (Geerligs, Rubinov, Cam-CAN, & Henson, 2015), that individual differences in personality can predict the effect of state-inductions on intrinsic resting-state connectivity (Servaas et al., 2013), and that the distinction between structural and functional connectivity is also relevant to the disentanglement of states and traits (Baur et al., 2013).

A third question is the synthesis of brain connectivity data, behavioral dispositions, and actual behavior. The vast majority of cognitive and personality network neuroscience studies still focuses on resting-state fMRI data and/or structural connectivity. More recently, however, studies have started to explore changes in the brain’s intrinsic functional architecture during behavior (Bolt, Nomi, Rubinov, & Uddin, 2017; Cohen & D’Esposito, 2016; Cole et al., 2014; Geerligs et al., 2015). One study that also included a personality measure identified a network cluster of changed connectivity within the limbic system during the processing of emotional facial expressions. Connectivity within this limbic network cluster showed a considerable test–retest reliability and correlated with trait anxiety (Cao et al., 2016). A complete personality network neuroscience account should not only describe neuropsychological trait systems in the resting brain but also demonstrate how these systems respond to stimulation and produce individual differences in behavior. Future research can either focus on brain network properties that have been linked to self-report personality scores, and test whether these network properties change during trait-relevant stimulation and behavior. Alternatively, brain regions with known functional implications for trait-relevant behavior can be studied from a network perspective. One example for such an endeavor would be the study of functional and structural connectivity between brain areas implicated in the anxiety/fear-related activation gradient from the periaqueductal gray to anterior areas of the cortex (Mobbs et al., 2009). Furthermore, we advocate to combine more real-life measures that go beyond self-report with neuroscientific data such as resting-state fMRI. In recent years, powerful smartphone technologies have started to allow researchers to record human behavior in everyday life (Markowetz, Błaszkiewicz, Montag, Switala, & Schlaepfer, 2014; Yarkoni, 2012). With the upcoming of the Internet of Things, the combination of digital traces of a person with neuroscientific data will become a natural research area at the interface between psychology, neuroscience, and computer science (“PsychoNeuroInformatics,” Montag et al., 2016). Of note, the feasibility to combine brain imaging data with smartphone tracked variables has been demonstrated recently (Montag et al., 2017).

Just like in other fields of psychology, a pressing issue of personality neuroscience is sample size, statistical power, and replicability of individual differences findings. Many MRI studies suffer from a lack of statistical power (Cremers, Wager, & Yarkoni, 2017) and individual differences analyses of functional connectivity data require large sample sizes (Kelly, Biswal, Craddock, Castellanos, & Milham, 2012). Collaborations between personality researchers across centers and open science data sharing efforts are needed to comprehensively tackle the relationship between personality and the human brain. Important first steps in this direction are currently underway (see Mendes et al., 2017, for the description of a large open science data set with a wealth of individual differences variables). In the cognitive neurosciences, the last years have seen the rise of large neuroinformatics platforms (Poldrack & Yarkoni, 2016). A similar strategy for individual differences research would be a desirable endeavor for future research.

Despite these open questions, network neuroscience approaches have also promising prospects that go beyond fundamental research on personality traits. An important application of connectomics in general and resting-state fMRI in particular is neuroimaging in populations that show lower levels of compliance to follow instructions during complex task protocols (Fox & Greicius, 2010). Resting-state and DTI protocols usually entail the minimum instruction to lie still for some minutes, which can be more easily followed by patients with psychiatric or neurodegenerative symptoms than complex behavioral task instructions. When sufficient precautions are taken to minimize confounding head motion (Power, Barnes, Snyder, Schlaggar, & Petersen, 2012), connectivity MRI can reveal neural correlates of personality traits in impaired patient populations. Personality traits can qualify as risk factors for or endophenotypes of psychiatric disorders (Benjamin, Ebstein, & Belmaker, 2001; Kendler, Neale, Kessler, Heath, & Eaves, 1993). A more detailed account of the neural implementation of personality traits could thus make important contributions to psychiatric research and the clinical neurosciences.

In order to grow to its full potential, the connectome paradigm will need to solve several methodological challenges. It has been shown that methodological details such as thresholding connectivity matrices and the resolution level of the whole brain parcellation scheme can bias results and interpretation (de Reus & van den Heuvel, 2013; van den Heuvel et al., 2017). Statistical issues include the risk of alpha-error inflation due to the immense amount of data in network maps and the formulation of appropriate null models of brain connectivity to evaluate the statistical significance of topological features of brain networks (Fornito, Zalesky, & Breakspear, 2013). Furthermore, biologically plausible and computationally inexpensive methods for the estimation of effective connectivity are needed, in order to assess the direction of functional connectivity estimates. And finally, data processing pipelines need to be optimized and unified to make the most of publicly available data sets and in order to ease replicability of findings across centers (Yan, Craddock, Zuo, Zang, & Milham, 2013).

A limitation of the present commentary is its narrow focus on non-ability trait aspects of personality that relate to affective and motivational behavior. Network aspects of individual differences in cognition are just as relevant for personality network neuroscience as the more narrow focus on personality taxonomies such as the Big Five. Several studies have addressed network aspects of ability traits such as working memory capacity, attentional ability, and general intelligence (Hilger, Ekman, Fiebach, & Basten, 2017; Markett et al., 2014, 2018; van den Heuvel, Stam, Kahn, & Hulshoff Pol, 2009). This research can also be relevant for understanding more cognitive aspects of the Big Five, such as conscientiousness, which is mostly uncorrelated with cognitive ability (but see Chamorro‐Premuzic & Furnham, 2004), but paradoxically related to similar brain regions (Allen & DeYoung, 2017).

Despite of the many open questions and the early stage of network neuroscience, we believe that this paradigmatic extension of our methodological toolbox will prove to be fruitful for our field. In a famous quote, personality psychologist Jeffrey Gray has positioned that “in the long run, any account of behaviour which does not agree with the knowledge of the nervous and endocrine systems which has been gained through the direct study of physiology *must* be wrong” (1987, p. 241). Research from the past 10 years has shown that the human brain is a network and that structural and physiological network properties are relevant to its function. Accounts of human personality that are not in line with this evidence will at best remain incomplete.

## Conflicts of Interest:

The authors have no conflicts of interest to declare.
